# Imitation learning based on an intrinsic motivation mechanism for efficient coding

**DOI:** 10.3389/fpsyg.2013.00800

**Published:** 2013-11-05

**Authors:** Jochen Triesch

**Affiliations:** Department of Neuroscience, Frankfurt Institute for Advanced StudiesFrankfurt, Germany

**Keywords:** intrinsic motivation, imitation, efficient coding, active perception, language development, bird song, mirror neuron, perceptual fluency

## Abstract

A hypothesis regarding the development of imitation learning is presented that is rooted in intrinsic motivations. It is derived from a recently proposed form of intrinsically motivated learning (IML) for efficient coding in active perception, wherein an agent learns to perform actions with its sense organs to facilitate efficient encoding of the sensory data. To this end, actions of the sense organs that improve the encoding of the sensory data trigger an internally generated reinforcement signal. Here it is argued that the same IML mechanism might also support the development of imitation when general actions beyond those of the sense organs are considered: The learner first observes a tutor performing a behavior and learns a model of the the behavior's sensory consequences. The learner then acts itself and receives an internally generated reinforcement signal reflecting how well the sensory consequences of its own behavior are encoded by the sensory model. Actions that are more similar to those of the tutor will lead to sensory signals that are easier to encode and produce a higher reinforcement signal. Through this, the learner's behavior is progressively tuned to make the sensory consequences of its actions match the learned sensory model. I discuss this mechanism in the context of human language acquisition and bird song learning where similar ideas have been proposed. The suggested mechanism also offers an account for the development of mirror neurons and makes a number of predictions. Overall, it establishes a connection between principles of efficient coding, intrinsic motivations and imitation.

## 1. Introduction

Imitation is a powerful form of learning where an agent acquires a skill from observing the skill being performed by a second agent. This can dramatically speed up the learning of useful behaviors compared to random exploration (Miller and Dollard, 1941). In the animal learning literature, imitation has been defined as “the copying of a novel or otherwise improbable act or utterance, or some act for which there is clearly no instinctive tendency” (Thorpe, 1963), but many other more or less stringent definitions exist. Many authors reserve the term imitation to situations where the behavior in question is not yet in the behavioral repertoire of the imitating agent (Clayton, 1978), but assessing the behavioral repertoire of an animal is in itself problematic. In the following, I will simply use imitation as an umbrella term for various forms of social learning and highlight important distinctions in the context of specific examples.

Despite many years of research, the origin and development of imitation abilities in animals and humans are still poorly understood (Heyes, 2001). While some theories have proposed that the ability to imitate relies on sophisticated innate mechanisms (Meltzoff and Moore, 1997), other accounts have emphasized the role of generic learning mechanisms for the development of imitative behaviors (Miller and Dollard, 1941; Gewirtz, 1969). Recent learning accounts considering possible underlying neurobiological mechanisms have rested on associative (Hebbian) learning (Heyes and Ray, 2000; Keysers and Perrett, 2004) or reinforcement learning (Triesch et al., 2007). These are sufficient for the development of a simple form of imitation also called *response facilitation*, where the agent learns to map the observation of a behavior performed by a second agent onto an already existing motor representation for performing the same behavior. This motor representation could already be present at birth or have been learned previously through random exploration of movement possibilities, often referred to as *babbling*. Importantly, however, these accounts have difficulties explaining the development of what is sometimes called *true imitation*, where the to-be-learned skill is not yet in the behavioral repertoire of the developing agent. This is the much more difficult and interesting case, because it addresses how imitation could accelerate the acquisition of novel skills.

An important example is speech acquisition, where the infant learns to produce utterances from her native language based on interactions with her caregivers. Infants are capable of statistical learning and readily discover statistical patterns of their native language, but also the social interaction with caregivers is critical for normal development of speech, see Kuhl (2004) for review. A closely related case is the acquisition of songs in certain species of song birds. This learning has been related to human language learning (Marler, 1970; Doupe and Kuhl, 1999) and is used as a model system for it. As early as 1773 it was shown that birds learn their song(s) from experience during development (Barrington, 1773). For example, male juvenile zebra finches usually learn to sing a song that closely resembles that of their father. The learning proceeds in two phases. During a first phase of purely sensory learning, the juvenile bird is suspected to form an auditory template of the father's (or other social tutor's) song (Baptista and Petrinovich, 1984; Konishi, 2010). During a second phase of sensory-motor learning, the bird learns to produce a song to match the learned template. Depending on the species, the sensory and sensory-motor phases may or may not overlap. Presently it is still unclear through what precise mechanisms the juvenile bird manages to better and better approximate the father's song. Here I discuss how a recently proposed intrinsically motivated learning (IML) mechanism for efficient coding in active perception might be generalized for this form of imitation learning. This suggests that principles of efficient sensory coding may be a foundation for song learning in birds and speech acquisition in humans.

Intrinsic motivations have recently come into focus as important driving forces in the development of complex behaviors (Baldassarre and Mirolli, 2013). While there is still much debate about the correct definition of intrinsic motivations (Baldassarre, 2011), the term is usually used when referring to behaviors such as play or other “curious” exploration of the environment that seem unrelated to any immediate “extrinsic” goal such as the acquisition of food. This *hypothesis* article does not propose any specific computational model nor does it present any empirical results. It is merely discussing the new hypothesis in the context of existing work. In the following, I briefly review a recently proposed form of IML for efficient sensory coding in active perception. Then I show how a generalization of this mechanism may account for the development of imitative behaviors. This also suggests a mechanism for the development of mirror neurons. Finally, I discuss predictions that the proposed mechanism makes.

## 2. Intrinsically motivated learning for efficient coding in active perception

The efficient coding hypothesis posits that sensory systems strive to encode sensory information in an efficient manner by exploiting the statistical structure and redundancies present in the sensory data (Attneave, 1954; Barlow, 1961). Since its first formulation, numerous aspects of sensory coding have been successfully explained in this context. This includes research on how early visual representations can be understood as adaptations to the statistics of natural images (Simoncelli and Olshausen, 2001) as well as related findings in the auditory (Smith and Lewicki, 2006) and olfactory (Perez-Orive et al., 2002) modalities. While this research program has been highly successful, it has typically neglected the active nature of perception. In particular, the statistics of sensory signals are a result of both the natural environment and the organism's behavior. This implies that the behavior of the organism and in particular the movement of the sense organs could be utilized to make the encoding of sensory information more efficient.

**Figure 1:**
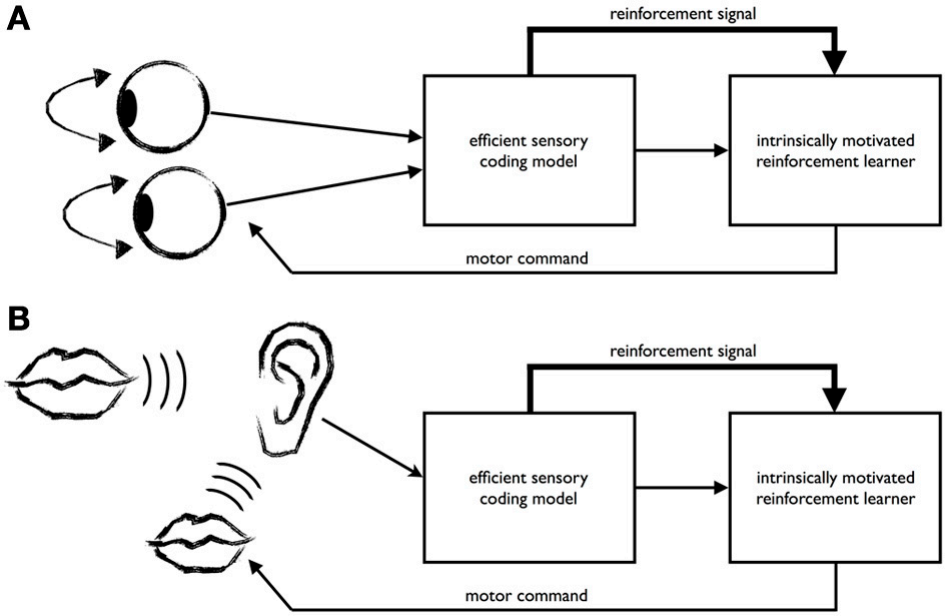
**The recently proposed intrinsically motivated learning architecture for efficient coding in active perception (A) also gives rise to the development of imitation (B). (A)** The learning architecture comprises an efficient coding model for the sensory input and an intrinsically motivated reinforcement learning mechanism for generating behavior. In the example of Zhao et al. (2012), the efficient coding model learns a sparse code for binocular images, while the reinforcement learner generates vergence eye movements. To this end, it receives from the sensory coding model a representation of the sensory input (thin arrow) and an internally generated reward signal reflecting how well the sensory model could encode the binocular input (thick arrow). Both the sensory coding model and the reinforcement learner try to optimize the encoding of the data. The system discovers that the input data can be encoded most efficiently when vergence commands are used to minimize binocular disparity. **(B)** The learner acquires an efficient encoding of speech signals provided by a tutor (big mouth). When the learner starts babbling (small mouth), the resulting acoustic signals are encoded by the sensory model that has been tuned to the tutor's speech. Signals that are easy to encode for the sensory model because the utterance sounds similar to the tutor's speech will produce a high reinforcement signal. Through this, the system's utterances are progressively driven to approximate the tutor's speech.

Along these lines and inspired by previous work from Schmidhuber (2009) proposing compression progress as an objective for IML, Zhao et al. (2012) have recently presented a model that learns to efficiently encode visual input from two eyes, see Figure 1A. Their approach proposes a form of IML using an internally generated reinforcement signal for learning efficient coding strategies in active perception. The method works as follows: A sensory model learns to encode sensory data, while a reinforcement learner generates actions of the sense organs that help the agent to encode the sensory data efficiently. To this end, an internally generated reinforcement signal is given to the reinforcement learner that reflects how well the sensory model is able to encode the input.

In the context of binocular vision Zhao et al. (2012) have shown that this mechanism elegantly explains the joint development of an efficient representation for stereo disparity in the sensory model and an accurate controller for vergence eye movements. In this setting, the system discovers that it is useful (intrinsically rewarding) to verge both eyes onto a common physical point, because then the sensory model is able to encode the data more efficiently. This is because the images from both eyes become more redundant and their joint encoding by the sensory model becomes more accurate. We may think of this in terms of the affordance concept. The observation of a certain disparity at the center of gaze is found to *afford* a certain vergence command that will lead to an improved representation of this input.

Importantly, the learning of the sensory model and the eye movement control develop jointly in this approach, driven by the identical objective of encoding the data efficiently. This mechanism has been shown to lead to fully autonomous and self-calibrating development of binocular vision and has been validated on a real robot (Lonini et al., 2013). More recently, it has also been extended to the development of smooth pursuit eye movements. Whether this approach can be extended to actions beyond eye movements is still an open question.

The central assumption of this approach is the existence of an internally generated reinforcement signal that encourages movements of the sense organs leading to an improved encoding of the sensory stimulus. Research on perceptual fluency supports the plausibility of this assumption. It has been found that the ease of processing of a sensory stimulus is related to positive affect (Reber et al., 1998). Assuming that the ease of processing reflects the quality of encoding of the stimulus by the sensory model, then easy to encode stimuli should produce positive affect. This positive affect may be due to the proposed internally generated reinforcement signal.

One point requires some discussion, however. Simply trying to behave such that the incoming sensory signals are encoded most easily might drive the agent to more or less abolish sensory input. In the case of visual perception, the agent could simply close the eyes or stare at a blank wall. This would make the sensory signals be encoded most easily, but is of little use otherwise. There are several ways to avoid this. A first solution is to introduce a separate mechanism for selecting *what* the agent will look at, while the described IML mechanism ensures that *how* the target object is being looked at is most efficient. For example, an attention mechanism selects what object in the scene should be looked at, while the proposed IML mechanism ensures that this particular object is well represented through vergence, smooth pursuit, and possibly other eye, head, and body movements. At the same time, it provides an optimized sensory encoding of the stimulus by properly taking into account the statistics of the sensory signals resulting from these movements. A second solution to the problem is to measure the ease of encoding of the sensory data in relation to some notion of the complexity of the data or the amount of information it contains. For example, the sensory signals resulting from staring at the blank wall may indeed be easy to encode (e.g., lead to a low reconstruction error of a generative model), but they may contain very little information to start with. There are various ways of making these notions mathematically precise, but the details are not important for the present paper.

Having introduced the recently proposed IML mechanism for efficient coding in active perception, we are now ready to consider its connection to imitation learning, which will require us to generalize it from movements of the sense organs to other motor acts.

## 3. How intrinsically motivated learning for efficient coding may support imitation

The mechanism for IML in active perception discussed above could also lead to the development of a form of imitation learning, as illustrated in Figure 1B. Consider the example of an infant faced with the problem of acquiring speech by imitating the utterances of her caregivers (or that of a juvenile song bird learning the father's song). Let's assume that at a certain point in development the infant has already learned a reasonably good sensory representation of what her native language sounds like Kuhl (2004). This representation will continue to improve with age and experience. When the infant vocalizes, her utterances will be processed by her own auditory system, which has already been tuned toward the sounds and words of her mother tongue. According to the IML mechanism described above, utterances that sound more like her mother tongue will be more easily encoded by her auditory system, which will lead to the generation of a higher reinforcement signal compared to utterances that sound dissimilar from her mother tongue. Thus, over time, the infant will adapt her utterances to the language she is exposed to driven by her intrinsic motivation to behave in such a way that the sensory data are encoded easily for her auditory system. Importantly, this suggests that language specific information could enter the babbling process early on, with each utterance being evaluated in the light of already learned sensory representations. We will return to this point in the Discussion.

An important question in this context is how the sensory model will learn to encode the caregiver's speech and when exactly the infant's speech will be easy to encode for the sensory model. The caregiver's utterances will necessarily sound different from the infant's utterances due to the different structure of their vocal tracts. For example, it is not lcear why the sound of a certain vowel produced by the infant with her vocal tract should be easy to encode for her auditory system, if this has been tuned to speech of her caregiver, whose vowels will generally differ in fundamental frequency and other parameters. For the case of vowel acquisition in the context of infant caregiver interactions, it has been argued that an *automirroring bias* can overcome this difficulty Ishihara et al. (2009); Miura et al. (2012).

### 3.1. Relative timing of sensory and motor learning

For The proposed IML mechanism it may be maladaptive for the learner to produce utterances at an excessive rate right after birth. If a sensory representation properly reflecting the correct target language (or song) is not acquired first, then the learner's auditory representation may become tuned to or even dominated by its own utterances. According to the proposed IML mechanism, the learner would then find rewarding whatever it is producing itself. This could potentially slow down learning of the native language. Enforcing a sufficient amount of passive exposure to the language may avoid this problem.

Similarly, reducing plasticity in sensory areas at the end of a critical period and before the onset of vocalizations may also alleviate this problem, because it prevents the sensory representation from becoming dominated by the sensory consequences of the agent's own actions.

An alternative solution to the problem would be to reduce or switch off sensory learning during one's own vocalizations. Instead, the auditory feedback could be used to train a forward model that predicts the auditory feedback based on an efference copy of the motor signals. Note that an accurate forward model allows planning and off-line learning without the need for producing actual motor output and observing the consequences. This can dramatically speed up learning (Sutton and Barto, 1998) and could even happen during sleep.

### 3.2. Learning one thing or many?

As discussed above, the absence of sensory input might be particularly easy to encode for the sensory system. This might lead the infant to not vocalize at all. Several solutions are conceivable. First, as suggested above the quality of encoding of the sensory model could be relative to the complexity of the sensory input or the amount of information contained in it. In this way, the situation of not babbling at all could be made comparatively undesirable. Second, a mechanism reinforcing the learning of novel cause and effect relationships or the “discovery of novel actions” (Redgrave and Gurney, 2006) could foster varied babbling. Third and maybe most obviously, the infant may want to communicate.

The question remains what and how many different things might be acquired through this IML mechanism. Note that while some bird species only learn a single song that “crystallizes” during development, others learn thousands of utterances during their life time (Catchpole and Slater, 2003) as do humans. If the sensory model allowed for only a single song “template” to be stored, this might explain why only a single song is learned. If, however, the sensory model had a high capacity for storing many acoustic patterns with high fidelity, then a large repertoire of actions would be learned with this mechanism. In general, for any kind of sensory model there will be a trade-off: given a fixed storage capacity more patterns can only be stored at the cost of storing them with smaller fidelity. Such differences could contribute to the varied vocabulary sizes in different species of song birds.

### 3.3. Context dependence

The mechanism described thus far will allow an agent to learn to imitate a range of utterances or behaviors whose sensory consequences match those of its learned sensory model. In the simplest case, however, all of these behaviors will appear equally “good” in any situation, i.e., what vocalization is performed would not necessarily depend on the current context. This could lead to behaviors being produced in inappropriate contexts. How could the agent learn to generate a certain behavior only in the appropriate context?

One solution is certainly through instrumental learning. If, say, the behavior has undesirable consequences in the present context, its execution may be made less probable because of this. A second solution to the problem is that during learning of the sensory model, contextual information is also integrated into the representation. Thus, the model will not be a purely sensory model anymore but a sensory-plus-context model. Specifically, if during the sensory-only phase of development, the infant or the song bird hears an utterance only in a specific context, then the developing sensory-plus-context model may encode this relationship. Thereby, if the learner generates the behavior in the same context, this will be particularly easy to encode for the sensory-plus-context model. Conversely, if the behavior is produced in a different context, this will be less easy to encode for the sensory-plus-context model, because there is a mismatch between the context and the sensory input. Obviously, relevant contexts are also perceived based on sensory, e.g., visual information. Thus a strict separation of sensory information and context may not always be possible. Interestingly, the context could be the presence of a certain object to which the infant pays attention. In this case, an initial association between the visual appearance of the object, it's acoustic label, and the motor representation for generating the acoustic label can be established. In this situation, the presense of the object would *afford* producing the object's name.

## 4. Development of mirror neurons

Mirror neurons are a class of neurons first observed in the premotor cortex of monkeys (Gallese et al., 1996) whose defining characteristic is that they can be activated if the monkey observes another agent performing a certain behavior or if the monkey plans and executes the same behavior. Because of this, they have been implicated in action understanding, imitation, empathy and language acquisition (Rizzolatti and Arbib, 1998; Gallese et al., 2004; Rizzolatti and Craighero, 2004). While originally discovered in monkeys, there is converging evidence for a mirror neuron system in humans (Iacoboni et al., 1999) and song birds (Prather et al., 2008). While the question how mirror neurons could support imitation has received much interest (Iacoboni et al., 1999; Iacoboni, 2005, 2009), comparatively little work has investigated how mirror neurons develop ontogenetically and what learning processes drive this development (Heyes, 2010).

Complementary mechanisms have been proposed for the development of mirror neurons based on generic learning principles. The most popular one is that mirror neurons develop through associative learning mechanisms such as Hebbian learning (Heyes and Ray, 2000; Keysers and Perrett, 2004; Heyes et al., 2005; Catmur et al., 2007; Cooper et al., 2013). A second mechanism is that mirror neurons could develop through reward-dependent (instrumental, reinforcement) learning (Triesch et al., 2007). We will take a look at both mechanisms before describing a new one based on IML for efficient coding, which combines aspects of the other two.

### 4.1. Hebbian development of mirror neurons

Hebbian accounts works as follows (Heyes and Ray, 2000; Keysers and Perrett, 2004; Del Giudice et al., 2009). In the case of behaviors whose sensory consequences are easily observed such as seeing one's own reaching movement or hearing one's own utterances, it is assumed that Hebbian learning forms associations between simultaneously active sensory and motor representations for already learned skills. As a result, neurons involved in the execution of a specific behavior receive strong excitatory connections from neurons representing its sensory consequences and vice versa. When another agent is then observed performing the same action, the same sensory representations will be triggered due to their ability to generalize to similar sensory stimuli. It has been argued that such generalization ability may stem from maturational constraints of the visual system Nagai et al. (2011). The activated sensory representation then excites the corresponding motor representation via the associative connections learned through the Hebbian mechanism. Through this the motor representation has obtained mirror properties: it is activated by planning or executing a behavior and by merely observing it in another agent.

The situation is more difficult for behaviors where the agent cannot fully perceive the sensory consequences of its actions as in the generation of facial expressions. For such “opaque” cases it is assumed that the agent learns to imitate by first being imitated by another agent—usually the caregiver. For example, when an infant smiles and his mother imitates the smile, the infant can learn to associate the visual representation of the mother's smiling face with her own motor representation for smiling. Again, the motor representation assumes mirror properties due to Hebbian learning. While overall the account appears plausible, a limitation is that it only develops mirror representations for skills that have already been learned. The learning of novel behaviors is left to random exploration which is very inefficient when many motor degrees-of-freedom are involved as is the case in speech or song production, i.e., when learning takes place in a high-dimensional space.

### 4.2. Reward-driven development of mirror neurons

In the reward-based learning account, the agent discovers that performing a certain behavior is useful whenever it sees another agent perform this behavior. For example, when a developing monkey observes a conspecific grasping a peanut from a source, the resulting sensory representation can become associated with the monkeys own motor plan for grasping a peanut from the same source, which is inherently rewarding—especially when hungry. Note that this mechanism does not require the ability to observe the sensory appearance of one's own action, but only whether it leads to a positive, i.e., reinforcing outcome. Circumstantial evidence for the importance of reward-driven learning in the development of mirror neurons comes from a recent finding that mirror neurons in monkey premotor area F5 are modulated by the value the monkey assigns to a grasped object (Caggiano et al., 2012).

The reward-driven account was studied in greatest detail in the context of gaze following, where an agent learns to look where others are looking. This is an example of a behavior where the sensory appearance of the behavior cannot be observed while the agent performs it. Triesch et al. (2007) proposed a computational model for the development of gaze following and showed that it produced mirror neurons for looking behaviors. It also explained various other aspects of the development of gaze following (Jasso et al., 2012). The existence of mirror neurons was the central prediction of the model and it was later confirmed neurophysiologically (Shepherd et al., 2009).

Interestingly, the reward-driven learning mechanism also predicts the possibility of generalized mirror neurons (Triesch et al., 2007). An agent may discover that it is useful to perform some action A whenever another agent is observed performing an action B. Gaze following represents a simple example of this: when two agents face each other, proper gaze following requires the learning agent to turn the head to his left if the model is observed turning the head to its right. Thus, not the physical appearance of the movement matters, but the goal of the action: where should I look? Through the reward driven learning mechanism an association can be learned from the sensory representation corresponding to the observation of the other agent performing action B and one's own motor representation of action A. This would lead to generalized mirror neurons for which the observed action triggering them is not necessarily identical to the action being generated.

### 4.3. Intrinsically motivated development of mirror neurons

The proposed IML mechanism integrates ideas from the Hebbian and the reward-based accounts. Like the Hebbian mechanism, it requires that the sensory consequences of the actions can be perceived. The development of mirror neurons could proceed along the following steps. (1) During sensory-only learning, a sensory model of various behaviors produced by the tutor is learned. Associated with this model, we assume that there will be populations of neurons specific to the perception of these different behaviors. (2) During the sensory-motor phase, the learner acquires motor representations that produce the same sensory consequences by virtue of the proposed IML mechanism. This involves the learner's reward system, but the reinforcement signals are internally generated. In the end, specific motor representations and the associated populations of neurons will code for specific behaviors. (3) Since these motor representations trigger specific sensory consequences, Hebbian learning mechanisms can establish a bidirectional association between the motor representation and the sensory representation. Through this, the sensory representation will acquire some motor properties and the motor representation will acquire some sensory properties. The clear distinction between sensory and motor representations dissolves and neurons with mirror properties develop: They are active when their sensory representation is triggered during observation of the behavior of another agent and during planning and execution of the corresponding behavior. Note that, the three steps could also overlap in time.

The computational benefit of the IML mechanism over the Hebbian mechanism is that the discovery of new skills is not left to random exploration, but occurs under guidance from the sensory model. Exploration is focused on those behaviors that produce similar sensory consequences as the behavior of conspecifics. The computational advantage over the reward-based mechanism is similar. The discovery of new skills does not require an external reward such as the peanut in the above example, but guarantees that matching one's behavior with that of a conspecific is intrinsically rewarding. This seems to better reflect the true nature of at least human imitation.

## 5. Discussion

I have described how a recently proposed mechanism for IML for efficient coding in active perception can be generalized to support imitative learning. In addition, a corresponding account for the development of mirror neurons was presented. It combines previous proposals based on associative Hebbian learning and instrumental or reinforcement learning in the framework of IML. These mechanisms represent parallel pathways through which mirror neurons can be acquired. Once established through either of these mechanisms, it is easy to see how mirror neurons could contribute to various forms of imitation including automatic imitation (Heyes, 2010) and vocal mimicry.

The IML mechanism proposed here is compatible with many previous theoretical accounts and computational models of song bird learning. A full review of these works is beyond the scope of this article. Existing works typically assume that a reinforcement signal is derived from matching auditory feedback to a stored sensory template (Doya and Sejnowski, 1995; Troyer and Doupe, 2000). Here I have proposed that such a reward signal could be derived from an evaluation of how well the auditory feedback is encoded by a sensory model. This distinction is admittedly subtle, but it connects the present approach to theories on efficient coding and sparse coding models as we have used in our work on the role of the same IML mechanism in active perception (Zhao et al., 2012; Lonini et al., 2013). This may be important, since neural representations in certain parts of the song system are known to be very sparse (Hahnloser et al., 2002).

The examples of human language acquisition and bird song learning are special in that the sensory consequences of the behavior are readily perceived. Obviously, the proposed mechanism can be extended to other actions that are easily perceived such as manual actions. For other actions such as facial expressions, this is not straight forward (unless a mirror is available). Learning to imitate facial expressions may require other mechanisms such as being imitated by caregivers (Heyes, 2001) or rely on reinforcement learning mechanisms and social feedback.

The presented mechanism is rooted in the efficient coding hypothesis. As such, it somewhat downplays the importance of social feedback during speech and song acquisition. But the social context in which learning takes place is known to play a very important role both in human language acquisition and bird song learning Goldstein et al. (2003); Kuhl et al. (2003). In the words of Goldstein and Schwade (2008): “infants' prelinguistic vocalizations, and caregivers' reactions to those immature sounds, create opportunities for social learning that afford infants knowledge of phonology.”

The proposed IML mechanism also shares some aspects of previous work on imitation in the developmental robotics literature. For instance, (Gaussier et al., 1998) and (Andry et al., 2001) propose a robot where a mechanism of “cognitive homeostasis” would give rise to imitative behaviors. Due to a “perceptual ambiguity” the robot may mistake an optic flow field caused by observing a moving agent with the flow field produced by its own locomotion. The homeostasis drive would try to minimize the mismatch between the sensory input stream and the robot's motor commands such that the robot will start moving. This is suggested to lead to an immediate following behavior. They then present experiments with a real robot that has a different prewired following mechanism. It learns to store extended sequences of movements resulting from following another robot or a human if these sequences lead to a reward. In our case, imitation does not emerge from a drive to reduce the mismatch between sensory percepts and own motor commands or from a prewired following mechanism but from a reinforcement signal that favors movements whose sensory consequences can be encoded efficiently by the sensory system.

Kaplan and Oudeyer (2007) have considered an intrinsic motivation for maximizing learning progress and discussed its potential role in the development of imitation. After illustrating how an intrinsic motivation for learning progress allows an agent to tackle progressively more difficult learning problems by discovering “progress niches,” they speculate that such an intrinsic motivation may also contribute to the development of imitation. Specifically, they argue that “(1) the meaningful distinctions necessary for the development of imitation (self, others and objects in the environment) may be the result of discriminations constructed during a progress-driven process and that (2) imitative behavior can more generally be understood as a way of producing actions in order to experience learning progress.” They speculate that at different stages of development infants may engage in different kinds of imitative behaviors because they maximize the infant's current learning progress. Here we argue that imitative behaviors are reinforced because their sensory consequences can be encoded efficiently by the learner's sensory model.

How could the proposed IML mechanism be tested experimentally? In the context of human language learning, it suggests that the babbling process might already reflect some aspects of the statistical properties of the language to which the infant has been exposed. This in turn predicts that the babbling process of infants could be shaped by carefully controlling their language input. For example, we may speculate that when caregivers intuitively reply to babbling attempts by uttering “close” words from the target language, they will affect the infant's sensory model in such a way that the correct pronunciation of the “close” word is reinforced during future babbling attempts. In contrast, replying to infant's babbling attempts with arbitrary different-sounding words will not produce this effect. Other aspects of child-directed speech such as hyperarticulation are also thought to aid the infant in learning a sensory model of the target language (Kuhl et al., 1997). More research is needed to investigate if and how infants' babbling is shaped by their developing sensory model of the target language through internally generated reinforcement signals.

In the context of bird song learning, the IML mechanism could be tested most directly by recording from reward circuits in the song bird brain as the animal is learning its song. The most obvious and direct prediction is that utterances sounding more similar to the father's song will generate a higher reward signal because they are easier to encode for the bird's auditory system, while utterances sounding dissimilar from the father's song will generate a lower reward signal because they are harder to encode. By manipulating the auditory feedback the bird is receiving, the causal role of this sensory feedback in learning can be tested. Note, however, that disentangling whether a stronger reinforcement signal is due to an easier encoding of the sensory signals or a greater similarity of the auditory feedback to a stored template may be difficult. To this end, it may be important to consider song bird species learning many different songs.

Next to testing the proposed mechanism and its possible neural implementation in biological experiments, it will also be interesting to apply the idea in the context of robots. For example, future work could try to exploit the proposed IML mechanism for language learning in robots. This will help to identify possible limitations or inconsistencies of the approach. The experiences gained would help to further develop and refine the current proposal. In conclusion, it is intriguing that the venerable principle of efficient sensory coding may play a central role in sophisticated cognitive phenomena such as imitation and language acquisition.

## Funding

This work was supported by the Quandt foundation, the European Communitys Seventh Framework Programme FP7/2007-2013, Challenge 2 - Cognitive Systems, Interaction, Robotics, under grant agreement No FP7-ICT-IP-231722, project IM-CLeVeR Intrinsically Motivated Cumulative Learning Versatile Robots, the DAAD through the Germany/Hong Kong Joint Research Scheme (project number G HK25/10), and the BMBF through Project Bernstein Fokus: Neurotechnologie Frankfurt, FKZ 01GQ0840.

### Conflict of interest statement

The authors declare that the research was conducted in the absence of any commercial or financial relationships that could be construed as a potential conflict of interest.

## References

[B1] Andry P. Gaussier P. Moga S. Banquet J.-P. Nadel J. (2001). Learning and communication via imitation: an autonomous robot perspective. Syst. Man Cybern. A 31, 431–442 10.1109/3468.952717

[B2] Attneave F. (1954). Some informational aspects of visual perception. Psychol. Rev. 61, 183 10.1037/h0054663 13167245

[B3] Baldassarre G. (2011). What are intrinsic motivations? A biological perspective, in Proceeding IEEE International Confereence on Development and Learning (ICDL), (Frankfurt), 1–8 10.1109/DEVLRN.2011.6037367

[B4] Baldassarre G. Mirolli M. (eds) (2013). Intrinsically Motivated Learning in Natural and Artificial Systems. Berlin: Springer 10.1007/978-3-642-32375-1

[B5] Baptista L. Petrinovich L. (1984). Social interaction, sensitive phases, and the song template hypothesis in the white-crowned sparrow. Anim. Behav. 32, 172–181 10.1016/S0003-3472(84)80335-8

[B6] Barlow H. B. (1961). Possible principles underlying the transformations of sensory messages. Chapter 13, in Sensory Communication, ed Rosenblith W. (M.I.T. Press), 217–234

[B7] Barrington D. (1773). Experiments and observations on the singing of birds. Philos. Trans. R. Soc. Lond. 63, 249–291 10.1098/rstl.1773.0031

[B8] Caggiano V. Fogassi L. Rizzolatti G. Casile A. Giese M. A. Thier P. (2012). Mirror neurons encode the subjective value of an observed action. Proc. Natl. Acad. Sci. U.S.A. 109, 11848–11853 10.1073/pnas.1205553109 22753471 PMC3406819

[B9] Catchpole C. K. Slater P. J. (2003). Bird Song: Biological Themes and Variations. Cambridge: Cambridge University Press

[B10] Catmur C. Walsh V. Heyes C. (2007). Sensorimotor learning configures the human mirror system. Curr. Biol. 17, 1527–1531 10.1016/j.cub.2007.08.006 17716898

[B11] Clayton D. (1978). Socially facilitated behavior. Q. Rev. Biol. 53, 373–391 10.1086/410789

[B12] Cooper R. P. Cook R. Dickinson A. Heyes C. M. (2013). Associative (not Hebbian) learning and the mirror neuron system. Neurosci. Lett. 540, 28–36 10.1016/j.neulet.2012.10.002 23063672

[B13] Del Giudice M. Manera V. Keysers C. (2009). Programmed to learn? The ontogeny of mirror neurons. Dev. Sci. 12, 350–363 10.1111/j.1467-7687.2008.00783.x 19143807

[B14] Doupe A. Kuhl P. (1999). Birdsong and human speech: common themes and mechanisms. Annu. Rev. Neurosci. 22, 567–631 10.1146/annurev.neuro.22.1.567 10202549

[B15] Doya K. Sejnowski T. (1995). A novel reinforcement model of birdsong vocalization learning. Adv. Neural Inf. Process. Syst. 7, 101–108

[B16] Gallese V. Fadiga L. Fogassi L. Rizzolatti G. (1996). Action recognition in the premotor cortex. Brain 119, 593–609 10.1093/brain/119.2.593 8800951

[B17] Gallese V. Keysers C. Rizzolatti G. (2004). A unifying view of the basis of social cognition. Trends Cogn. Sci. 8, 396–403 10.1016/j.tics.2004.07.002 15350240

[B18] Gaussier P. Moga S. Quoy M. Banquet J.-P. (1998). From perception-action loops to imitation processes: a bottom-up approach of learning by imitation. Appl. Artif. Intell. 12, 701–727 10.1080/088395198117596

[B19] Gewirtz J. L. (1969). Mechanisms of social learning: Some roles of stimulation and behavior in early human development. Handbook Soc. Theory Res. 57–212 21894151

[B20] Goldstein M. H. King A. P. West M. J. (2003). Social interaction shapes babbling: testing parallels between birdsong and speech. Proc. Natl. Acad. Sci. U.S.A. 100, 8030–8035 10.1073/pnas.1332441100 12808137 PMC164707

[B21] Goldstein M. H. Schwade J. A. (2008). Social feedback to infants' babbling facilitates rapid phonological learning. Psychol. Sci. 19, 515–523 10.1111/j.1467-9280.2008.02117.x 18466414

[B22] Hahnloser R. H. Kozhevnikov A. A. Fee M. S. (2002). An ultra-sparse code underlies the generation of neural sequences in a songbird. Nature 419, 65–70 10.1038/nature00974 12214232

[B23] Heyes C. (2001). Causes and consequences of imitation. Trends Cogn. Sci. 5, 253–261 10.1016/S1364-6613(00)01661-2 11390296

[B24] Heyes C. (2010). Where do mirror neurons come from? Neurosci. Biobehav. Rev. 34, 575–583 10.1016/j.neubiorev.2009.11.007 19914284

[B25] Heyes C. Bird G. Johnson H. Haggard P. (2005). Experience modulates automatic imitation. Cogn. Brain Res. 22, 233–240 10.1016/j.cogbrainres.2004.09.009 15653296

[B26] Heyes C. M. Ray E. D. (2000). What is the significance of imitation in animals? Adv. Study Behav. 29, 215–245 10.1016/S0065-3454(08)60106-05377343

[B27] Iacoboni M. (2005). Neural mechanisms of imitation. Curr. Opin. Neurobiol. 15, 632–637 10.1016/j.conb.2005.10.010 16271461

[B28] Iacoboni M. (2009). Neurobiology of imitation. Curr. Opin. Neurobiol. 19, 661–665 10.1016/j.conb.2009.09.008 19896362

[B29] Iacoboni M. Woods R. P. Brass M. Bekkering H. Mazziotta J. C. Rizzolatti G. (1999). Cortical mechanisms of human imitation. Science 286, 2526–2528 10.1126/science.286.5449.2526 10617472

[B30] Ishihara H. Yoshikawa Y. Miura K. Asada M. (2009). How caregiver's anticipation shapes infant's vowel through mutual imitation. Auton. Mental Dev. IEEE Trans. 1, 217–225 10.1109/TAMD.2009.2038988

[B31] Jasso H. Triesch J. Deák G. Lewis J. (2012). A unified account of gaze following. IEEE Trans. Auton. Mental Dev. 4, 257–272 10.1109/TAMD.2012.2208640

[B32] Kaplan F. Oudeyer P.-Y. (2007). The progress-drive hypothesis: an interpretation of early imitation, in Models and Mechanims of Imitation and Social Learning: Behavioural, Social and Communication Dimensions, eds Nehaniv C. Dautenhahn K. (Cambridge: Cambridge University Press), 361–377

[B33] Keysers C. Perrett D. (2004). Demystifying social cognition: a Hebbian perspective. Trends Cogn. Sci. 8, 501–507 10.1016/j.tics.2004.09.005 15491904

[B34] Konishi M. (2010). From central pattern generator to sensory template in the evolution of birdsong. Brain Lang. 15, 18–20 10.1016/j.bandl.2010.05.001 20955898

[B35] Kuhl P. K. (2004). Early language acquisition: cracking the speech code. Nat. Rev. Neurosci. 5, 831–843 10.1038/nrn1533 15496861

[B36] Kuhl P. K. Andruski J. E. Chistovich I. A. Chistovich L. A. Kozhevnikova E. V. (1997). Cross-language analysis of phonetic units in language addressed to infants. Science 277, 684–686 10.1126/science.277.5326.684 9235890

[B37] Kuhl P. K. Tsao F.-M. Liu H.-M. (2003). Foreign-language experience in infancy: effects of short-term exposure and social interaction on phonetic learning. Proc. Natl. Acad. Sci. U.S.A. 100, 9096–9101 10.1073/pnas.1532872100 12861072 PMC166444

[B38] Lonini L. Zhao Y. Chandrashekhariah P. Shi B. E. Triesch J. (2013). Autonomous learning of active multi-scale binocular vision, in Development and Learning (ICDL), 2013 IEEE International Conference on, (Osaka), 1–6

[B39] Marler P. (1970). Birdsong and speech development: Could there be parallels? There may be basic rules governing vocal learning to which many species conform, including man. Am. Sci. 58, 669–673 5480089

[B40] Meltzoff A. N. Moore M. K. (1997). Explaining facial imitation: a theoretical model. Early Dev. Parent. 6, 179–192 10.1002/(SICI)1099-0917(199709/12)6:3/4<179::AID-EDP157>3.0.CO;2-R PMC3953219 24634574

[B41] Miller N. E. Dollard J. (1941). Social Learning and Imitation. New Haven, CT: Yale University Press

[B42] Miura K. Yoshikawa Y. Asada M. (2012). Vowel acquisition based on an auto-mirroring bias with a less imitative caregiver. Adv. Robot. 26, 23–44 10.1163/016918611X607347

[B43] Nagai Y. Kawai Y. Asada M. (2011). Emergence of mirror neuron system: immature vision leads to self-other correspondence, in Development and Learning (ICDL), 2011 IEEE International Conference on, (Frankfurt), 1–6

[B44] Perez-Orive J. Mazor O. Turner G. C. Cassenaer S. Wilson R. I. Laurent G. (2002). Oscillations and sparsening of odor representations in the mushroom body. Science 297, 359–365 10.1126/science.1070502 12130775

[B45] Prather J. F. Peters S. Nowicki S. R. M. (2008). Precise auditory-vocal mirroring in neurons for learned vocal communication. Nature 451, 305–310 10.1038/nature06492 18202651

[B46] Reber R. Winkielman P. Schwarz N. (1998). Effects of perceptual fluency on affective judgments. Psychol. Sci. 9, 45–48 10.1111/1467-9280.000089521834

[B47] Redgrave P. Gurney K. (2006). The short-latency dopamine signal: a role in discovering novel actions? Nat. Rev. Neurosci. 7, 967–975 10.1038/nrn2022 17115078

[B48] Rizzolatti G. Arbib M. A. (1998). Language within our grasp. Trends Neurosci. 21, 188–194 10.1016/S0166-2236(98)01260-0 9610880

[B49] Rizzolatti G. Craighero L. (2004). The mirror-neuron system. Annu. Rev. Neurosci. 27, 169–192 10.1146/annurev.neuro.27.070203.144230 15217330

[B50] Schmidhuber J. (2009). Driven by compression progress: a simple principle explains essential aspects of subjective beauty, novelty, surprise, interestingness, attention, curiosity, creativity, art, science, music, jokes, in Anticipatory Behavior in Adaptive Learning Systems, (Springer), 48–76

[B51] Shepherd S. V. Klein J. T. Deaner R. O. Platt M. L. (2009). Mirroring of attention by neurons in macaque parietal cortex. Proc. Natl. Acad. Sci. U.S.A. 106, 9489–9494 10.1073/pnas.0900419106 19470477 PMC2685741

[B52] Simoncelli E. P. Olshausen B. A. (2001). Natural image statistics and neural representation. Annu. Rev. Neurosci. 24, 1193–1216 10.1146/annurev.neuro.24.1.1193 11520932

[B53] Smith E. C. Lewicki M. S. (2006). Efficient auditory coding. Nature 439, 978–982 10.1038/nature04485 16495999

[B54] Sutton R. S. Barto A. G. (1998). Reinforcement Learning: An Introduction. Cambridge, MA: Cambridge University Press

[B55] Thorpe W. (1963). Learning and Instinct in Animals. 2nd Edn Cambridge, MA: Harvard University Press

[B56] Triesch J. Jasso H. Deák G. O. (2007). Emergence of mirror neurons in a model of gaze following. Adapt. Behav. 14, 149–165 10.1177/1059712307078654

[B57] Troyer T. W. Doupe A. J. (2000). An associational model of birdsong sensorimotor learning I. Efference copy and the learning of song syllables. J. Neurophysiol. 84, 1204–1223 10979996 10.1152/jn.2000.84.3.1204

[B58] Zhao Y. Rothkopf C. A. Triesch J. Shi B. E. (2012). A unified model of the joint development of disparity selectivity and vergence control, In Development and Learning (ICDL), 2012 IEEE International Conference on, (San Diego, CA), 1–6

